# Effects of Dielectric Barrier Ambient Air Plasma on Two Brassicaceae Seeds: *Arabidopsis thaliana* and *Camelina sativa*

**DOI:** 10.3390/ijms22189923

**Published:** 2021-09-14

**Authors:** Maxime Bafoil, Mohammed Yousfi, Christophe Dunand, Nofel Merbahi

**Affiliations:** 1 Laboratoire Plasma et Conversion d’Energie (LAPLACE), Université Toulouse III Paul Sabatier, UMR CNRS 5213, 31062 Toulouse, France; bafoil@laplace.univ-tlse.fr (M.B.); merbahi@laplace.univ-tlse.fr (N.M.); 2 Laboratoire de Recherche en Sciences Végétales, Université de Toulouse, CNRS, UPS, Toulouse INP, 31326 Auzeville-Tolosane, France

**Keywords:** low-temperature ambient air plasma, plasma-agriculture, stimulated germination rate and speed, *Arabidopsis thaliana* and *Camelina sativa*, Brassicaceae seeds

## Abstract

We investigated low-temperature plasma effects on two Brassicaceae seeds (*A. thaliana* and *C. sativa*) using dielectric barrier discharge in air. Comparisons of plasma treatments on seeds showed distinct responses on germination rate and speed. Optimal treatment time giving optimal germination is 15 min for *A. thaliana* with 85% increase compared to control after 48 h of germination and 1 min for *C. sativa* with 75% increase compared to control after 32 h of germination. Such germination increases are associated with morphological changes shown by SEM of seed surface. For better understanding at the biochemical level, seed surfaces were analyzed using gas chromatography-mass spectrometry which underlined changes of lipidic composition. For both treated seeds, there is a decrease of saturated (palmitic and stearic) fatty acids while treated *C. sativa* showed a decrease of unsaturated (oleic and linoleic) acids and treated *A. thaliana* an increase of unsaturated ones. Such lipid changes, specifically a decrease of hydrophobic saturated fatty acids, are coherent with the other analyses (SEM, water uptake and contact angle). Moreover, an increase in *A. thaliana* of unsaturated acids (very reactive) probably neutralizes plasma RONS effects thus needing longer plasma exposure time (15 min) to reach optimal germination. For *C. sativa*, 1 min is enough because unsaturated linoleic acid becomes lower in treated *C. sativa* (1.2 × 10^7^) compared to treated *A. thaliana* (3.7 × 10^7^).

## 1. Introduction

In agronomy, the use of synthetic phytosanitary products allowing the improvement of seed germination, plant growth and also seed decontamination is a common practice [1]. However, in order to respect the environment and to control soil pollution, researchers are investigating the use of less harmful products. In this context, the research on the use of low-temperature plasmas in agriculture appears to be an interesting and promising alternative, particularly to improve the efficiency of the seed germination step.

Germination is a key stage in plant life. As defined by Bewley and Black, germination “brings together the events which result from the absorption of water by the dry seed at rest and end with the lengthening of the embryonic axis” [2]. Germination depends on many factors regardless of whether they are internal to the seed physiology, such as the age of the seed or the hormonal status, or external factors such as climatic conditions or soil and microbiota compositions [3]. The direct seed environment must be favorable throughout germination to ensure proper and rapid seedling emergence and plant establishment. However, in nature, environmental conditions can be favorable only for a very short time. In general, the acceleration of the germination rate will be an important if not necessary asset for optimal plant development and moreover the survival of the species thanks to high seed production in the case of healthy plants.

Low-temperature plasmas are generally produced at a temperature below about forty degrees due to their low ionization degree since only electrons are hot (or energetic) while the remaining heavy species generally remain close to the background gas temperature. Therefore, those “cold” plasmas are suitable for interactions with living tissues and organisms without significant thermal effects. The associated physio-chemical plasma properties (electric field, charged particles, photons and reactive species, more particularly those of oxygen and nitrogen species (RONS) are exploited in many biological applications such as (i) surfaces sterilization or decontamination due to the plasma bactericide effects [4], (ii) cell death in cancerology [5], (iii) cell regeneration in chronic wound healing [6], (iv) chemical etching for cell membrane permeabilization in gene transfection [7]. Low temperature plasmas have been used also in agriculture with interesting improvements of the germination efficiency in seeds of various kinds. The effects on seed germination were observed using different plasma devices and a large variety of background gas compositions [8].

The association of researchers in plant biology and plasma physics dates back about 30 years with the pioneer work of Krapivina et al. [9]. After that, numerous studies have shown that cold plasma treatment can stimulate both the germination and plant growth of many seeds of agronomic interest (e.g., [8,10,11]).

For example, Sera et al. investigated the effect of a very short treatment time on the germination of sugar beet seeds (*Beta vulgaris*) using a coplanar plasma device [12]. Plasma treatment showed a positive effect as a function of treatment time. Interestingly, very short times for treatment, such as 1 s, are sufficient to increase by more than 20% the germination rate [12]. They have also shown that times of 30 s negatively affect the germination of these seeds.

It is also shown (e.g., [13]) on several parameters of seed germination of *Lens culinaris* seeds the effects of the plasma treatment generated by a dielectric barrier discharge (DBD) in helium with a small admixture of oxygen or nitrogen. It is reported that the positive effect on germination is due to changes on the seed surface, such as the increase in hydrophilicity, which is monitored, as usually done in the literature, thanks to the change of the contact angle between a water droplet and the seed surface, or even a better water uptake [13].

In another study [14], the onion seed germination treated with a plasma jet of helium and nitrogen powered by a radio frequency device was investigated. The response of the germination is an expected dependent of the treatment time since 15 s of treatment has shown a slightly negative effect while a good positive effect on germination is observed after treatment of 240 s (going from 91 to 99% maximum germination) [14].

However, most of these literature studies are mainly devoted to the observation of the increase or change of the germination rate for different treated seeds by different cold plasma devices while only a few of these studies tried to emphasize the plasma-induced mechanisms on seed germination.

For instance, it was noted that the positive effect of plasma treatment on the germination of soybeans (*Glycine max*) (up to +20%) could be due to the effect of a stress response [15]. Indeed, stimulation of enzymatic activity such as proteases, amylases and glucanases which are essential for germination and the mobilization of carbohydrate resources were observed after plasma treatment [15]. In the same study, the phenomenon of plasma dose-dependence with deleterious effects of a higher dose was demonstrated. Some DNA damage caused by plasma treatments was observed but without demonstrated importance [15].

However, the literature research works devoted to mechanisms at the cellular scale remains insufficient to understand well the chemical and physio-biological effects of the plasmas on the seeds. Therefore, more research works are needed to better explain the plasma-induced mechanisms on seed germination.

The aim of the present work is to contribute to a better understanding of the low temperature plasma effects on germination by choosing two Brassicaceae seeds *(Arabidopsis thaliana* and *Camelina sativa*). *A. thaliana* was considered as the model in plant biology for many years [16,17]. Indeed, its small size and short generation time as well as its large number of seeds are compatible with laboratory conditions. Moreover, the model plant has a small genome which is already fully sequenced. In addition, the lack of agronomic interest in this plant allows the dissemination of research to the scientific community. *C. sativa* is a seed with great agricultural potential mainly known for its high oil content. It belongs as *A. thaliana* to the Brassicaceae family. This allows data transfer between the two species and will make it possible to highlight the changes undergone after plasma treatment. In the case of *A. thaliana* treated with ambient air plasma, these previous studies have shown more positive effects on germination speed and plant development [18,19]. They have also allowed to set up the optimal protocol for germination assays using the present air plasma.

In this work, germination of both *A. thaliana* and *C. sativa* will be observed and analyzed to determine first the optimal processing time for the two seeds. Subsequently, the effects of plasma treatment on the seed surfaces are investigated. Then, the permeability of the seeds to tetrazolium salts was tested. This was followed by the determination of the contact angle on *C. sativa* seeds to underline the effect of plasma treatment on surface hydrophilicity. Then, the surface of the two kinds of seeds was analyzed using scanning electron microscopy (SEM) to observe the changes of the surface shape and the differences between the two treated seed surfaces and to compare them with the control seeds. Finally, from a bio-chemical point of view, the lipid composition of the seed surfaces is quantified and analyzed after plasma treatment using Gas Chromatography and Mass Spectroscopy (GC/MS) thus introducing some correlations between fatty acids change and germination improvement.

## 2. Results

### 2.1. Plasma Treatment Increases Seeds Germination in Time Exposure-Dependent Manner

**Figure 1:**
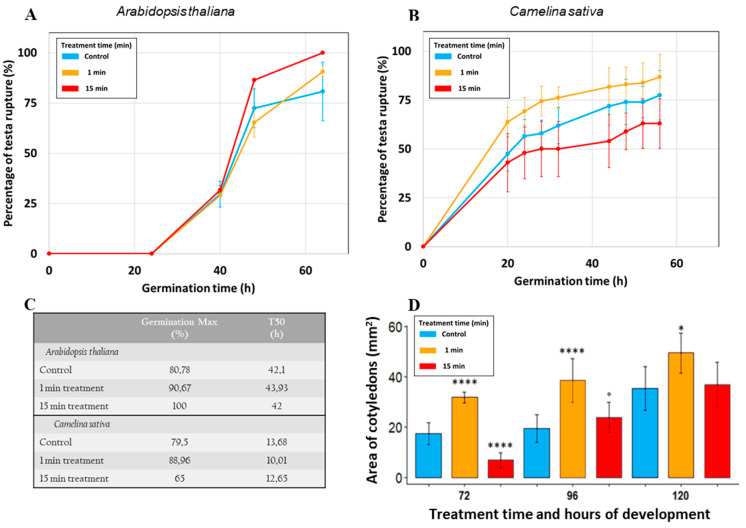
Germination parameters of *A. thaliana* and *C. sativa* seeds after two exposure times (1 and 15 min) to DBD air plasma with the comparisons to control seeds. Graph A shows the testa rupture of *A. thaliana* seeds and graph B shows the testa rupture of *C. sativa*. (**A**,**B**): The testa rupture is evaluated as a function of the development time for different treatment times (0, 1 and 15 min). The blue curves represent the control seeds, the orange curve the 1 min plasma-treated seeds and the red the 15 min-treated seeds (**C**): Maximum of germination (Gmax) for each seed and treatment time, and the T50 which is the time (in hours) needed to reach half of the Gmax. (**D**): Area of *C. sativa* cotyledons. The establishment of the *C. sativa* cotyledons is noted with pictures of germinated seeds and image analysis which allows the measurement of the area of the cotyledons. Those measurements were done on 10 Petri dishes at least three different times. The significance deviations were determined using the Kruskal–Wallis tests between the control and plasma-treated seeds: °: *p*-value < 0.1, *: *p*-value < 0.05, ****: *p*-value < 0.00005.

The effects of different plasma processing (or plasma exposure) times on seeds of *A. thaliana* and *C. sativa* are displayed in Figure 1. First, the percentage of testa rupture versus the development time for two processing times and control is monitored for the seeds of the two species.

The germination rate of *A. thaliana* seeds treated during 15 min with plasma shows an increase compared to the germination of the non-treated seeds from about 72% to 87%, 48 h after germination (*p*-value = 0.0377). However, a reduction in germination rate was observed for the seeds treated 1 min (from about 72% without treatment to 65%, Figure 1A). The germination rate increases for *C. sativa* seeds treated 1 min compared to the control seeds (from 62% to about 76% at 32 h after germination, Figure 1B). The germination rate decreases in the case of a 15-min treatment with DBD air plasma going from a final germination of about 80% for the controls to 65% after a 15-min treatment. Therefore, for both kinds of seeds, the plasma effect on the germination rate is time-treatment dependent.

To compare the plasma effect on the two kinds of seeds, a maximum of germination (Gmax) and T50 were reported (table of Figure 1C). Gmax is the maximal percentage of germination at the end of the kinetics of observations. The T50 corresponds to the time required to reach 50% of maximum germination and was obtained using a trend curve made with the seed germination points, and the equation of each curve solved with f(x) = Gmax/2.

Gmax of *A. thaliana* increases regardless of the treatment time but T50 is increased for a 1-min treatment compared to control seeds (respectively 43.9 and 42.1 h) meaning that the 1min treatment increases the germination rate but decreases the speed of germination. For the 15-min treatment, Gmax is increased until 100% and T50 is slightly reduced, which makes this treatment time optimal for *A. thaliana* with the present DBD air plasma set up.

Gmax of *C. sativa* is maximum at 1 min and decreases beyond 1-min treatment; this means that a treatment longer than 1 min has a negative effect on germination. For the 1-min treatment, the T50 to reach Gmax is shorter compared to the control by 3.67 h less. The cotyledon establishment of *C. sativa* seeds is also displayed (Figure 1D). The cotyledon area with or without plasma treatment is measured as a function of germination development time. For each measurement, it is observed that the 1 min plasma treatment with DBD plasma increases the size of the cotyledons compared to the control, an increase of +14.4 mm^2^ is reached at 72 h. There was also some reduction of cotyledon size after exposure of the seeds to plasma during 15 min at 72 h and no significant differences at 120 h (35.3 mm^2^ and 36.6 mm^2^).

### 2.2. Plasma Treatment Affects Seed Surface Structure

#### 2.2.1. Seed Morphology and Anatomy

**Figure 2:**
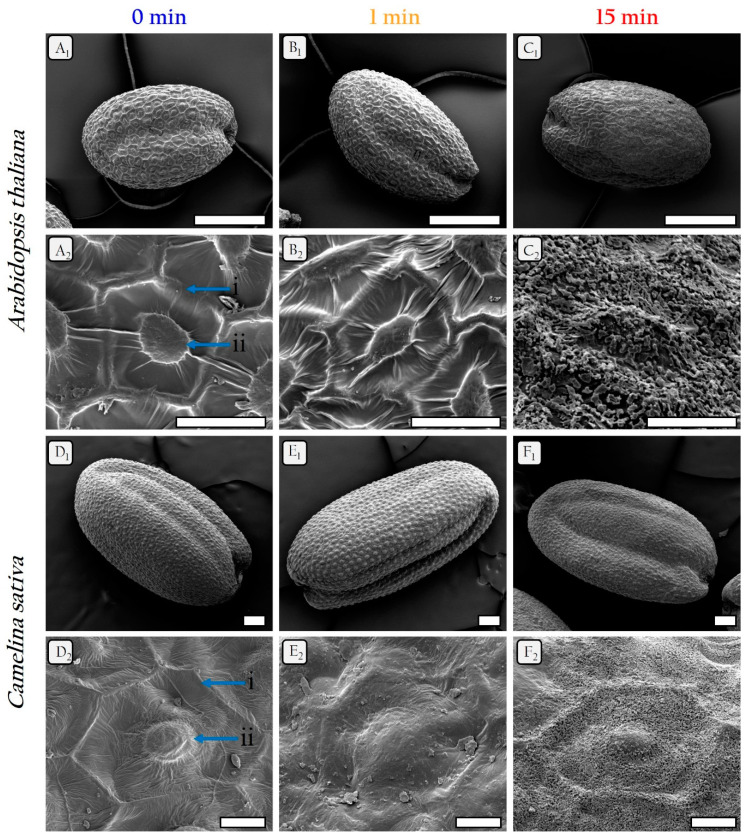
Scanning electron microscopy pictures of the seed surface of *A. thaliana* and *C. sativa* treated with plasma for two exposure times (1 min and 15 min) and compared to the control (0 min). The plasma treatment has an increased effect on the surface smoothing that therefore depends on the treatment time. The pictures (**A**–**C**) correspond to *A. thaliana* seeds. The pictures (**D**–**F**) correspond to *C. sativa*. In the first column, the seeds are not treated (**A**,**D**); In the second column, the seeds are exposed to the plasma for 1 min (**B**,**E**); And in the third column (**C**,**F**) the treatment time is 15 min. For the pictures of whole seeds (1) the scale bars are 200 µm, and for the pictures of one cell (2) the scale bars are 20 µm. i: radial cell wall, ii: columella.

Surfaces of *A. thaliana* and *C. sativa* seeds treated with plasma were observed by SEM (Figure 2). After 1 min of treatment, the surfaces of the treated seeds are slightly modified. However, after 15 min, there is a reduction in the shape of the columella as well as an accumulation of an unknown compound on the surface of the treated seeds, which results in some specific effect on this surface (Figure 2C,F). This effect, which looks like a physicochemical etching of the surface by plasma treatment, indicates the presence of a macromolecular structure probably rearranged with respect to the control. In addition, it has the appearance of a molten wax which cannot be attributed to the plasma temperature that does not exceed about 40 °C in the case of the present low temperature plasma treatment. This molten wax can be induced by chemical interactions as those involving RONS and/or physical interactions as those involving charged particles and electric field with the external layers of the seed surface as partly suggested by Pawlat et al. [20].

#### 2.2.2. Seeds Wettability

**Figure 3:**
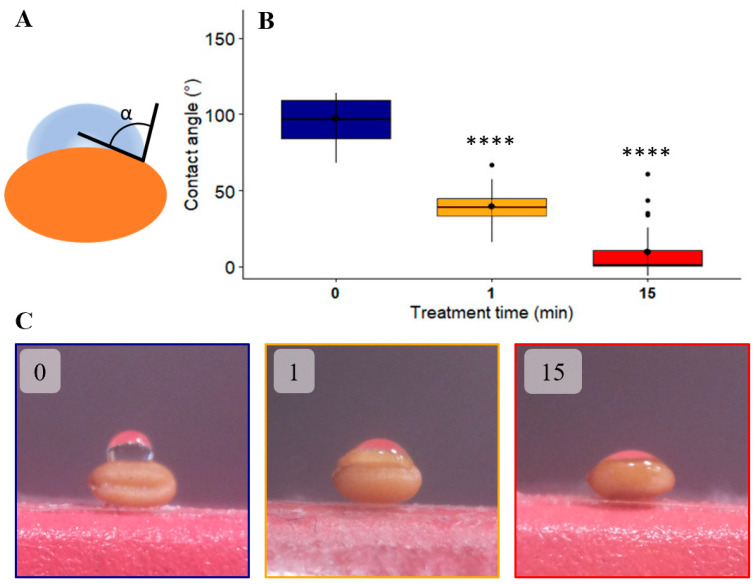
Contact angle assay on *C. sativa* seeds for different plasma treatment times. The amount of 0.3 µL of water is dropped on the surface of the *C. sativa* seeds and the contact angle is measured. (**A**): The scheme of the contact angle that is measured in this experiment. (**B**): Boxplot of the measurement of contact angle versus different treatment times. The seeds were treated with DBD air plasma for 1 min (orange), 15 min (red) or non-treated (blue). (**C**): Pictures of the water droplet on seeds with or without plasma treatment. The experiment is carried out on 30 seeds from 2 different exposures for each treatment (1 min and 15 min while 0 min corresponds to the control). The significance differences were determined using the Duncan test: ****: *p*-value < 0.00005.

*A. thaliana* seeds are too small to be used to determine the contact angle of a water droplet on the seed surface. Therefore, the contact angle was measured only for seeds of *C. sativa* (Figure 3A). The smaller the angle, the more the drop is spread over the surface of the seed and therefore the more hydrophilic the surface of the seed (Figure 3C). The distribution of the angle measurements is very homogenous independent of the plasma treatment time. The wettability of the seed surface treated with plasma is already significantly increased after 1 min of plasma treatment. After 15 min, the contact angle is 10 times reduced going from 96.9° to 9.3° (Figure 3B), which indicated an almost complete wettability of the seed.

#### 2.2.3. Permeability Tests

**Figure 4:**
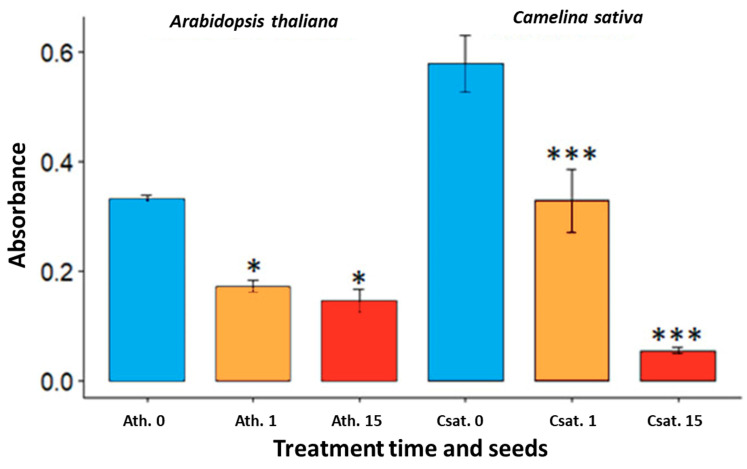
Bar plots of permeability tests with tetrazolium red on *A. thaliana* and *C. sativa* seeds after different plasma treatment times. Seeds were incubated for 24 h in tetrazolium red at 28 °C and the production of formazan is noted by measuring the absorbance of the incubation solution at 492 nm. The blue bars represent the control samples, the orange curves the air plasma treated seeds during 1 min and the red ones the 15 min treated seeds. On the left side, the absorbance for the *A. thaliana* seeds (Ath. 0, 1 and 15 min), and on the right the absorbance for the *C. sativa* seeds (Csat. 0, 1 and 15 min). The significant differences were determined using the Wilcoxon test between the control and plasma treated seeds: *: *p*-value < 0.01 ***: *p*-value < 0.001.

The permeability of the *A. thaliana* and *C. sativa* seeds treated or not treated with the DBD air plasma is quantified. The permeability could be noted with the presence of tetrazolium red which via an oxidized process turns red when it enters a cell. The quantification of the red staining reflects the seed permeability. The plasma treatments reduce the permeability of both seeds (Figure 4). For *A. thaliana*, there is an important decrease of permeability after just 1 min of treatment (from 0.33 to 0.17) and the decrease continues slowly, to reach after 15 min treatment, 0.14 (*p*-value = 0.009). For the seeds of *C. sativa*, a similar evolution can be observed with an important reduction after plasma treatment regarding the exposure times of 0, 1 and 15 min, since the corresponding absorbances are 0.58, 0.31 and 0.05 respectively. We will see in the discussion section that such a permeability property to tetrazolium salt can allow an increase of the germination rate of the treated seed even in a saline medium.

#### 2.2.4. Water Uptake

**Figure 5:**
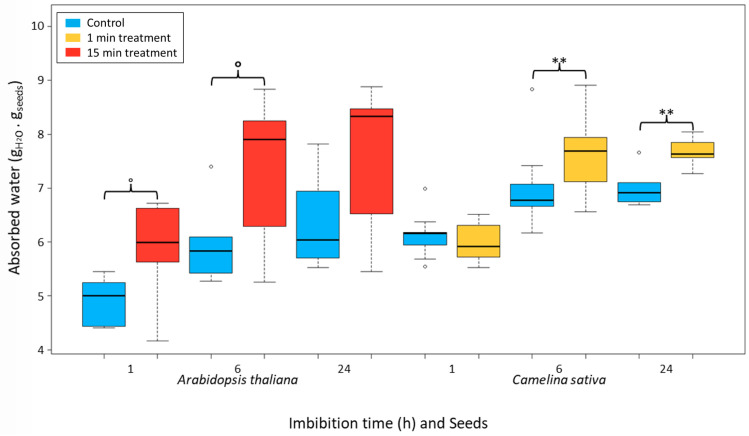
Bar plots of imbibition tests on *A. thaliana* and *C. sativa* seeds after 1, 6 or 24 h with or without plasma treatment. Between 0.02 and 0.04 g of seeds are placed in tubes in the presence of 1 mL of deionized water and stirred continually. The water is removed, and the seeds are weighed at 1 h, 6 h or 24 h after the beginning of the imbibition. The blue bars represent the control seeds, the red ones the treated seeds of *A. thaliana* (15 min of plasma treatment) while the orange bars the *C. sativa* seeds (1 min of plasma treatment). The weight of water absorbed is calculated regarding the weight of the imbibed seeds compared to the dry seeds. The importance of the differences was determined using the Wilcoxon test between the control and plasma-treated seeds: **: *p*-value < 0.005; ° *p*-value < 0.1.

The *A. thaliana* and *C. sativa* seeds are both imbibed with water and weighed after 1, 6 and 24 h. After 1 h of imbibition, for *C. sativa* there is no difference between the control and the treated seeds while for *A. thaliana*, a difference is observed between treated and control seeds (*p*-value of Wilcoxon test: 0.0649). Then, after 6 h of imbibition, *C. sativa* seeds with a 1 min plasma treatment showed a better imbibition than the control ones (11% more) with an increase of 0.74 g of H_2_O per g of dry seeds (*p*-value of Wilcoxon test: 0.0145). For *A. thaliana*, after 15 min of treatment, imbibition of treated seeds is better (24% more) with an increase of 1.43 g of H_2_O per g of dry seeds (*p*-value of Wilcoxon test: 0.0931). Last, after 24 h of imbibition, there is still an increase of imbibition for the plasma-treated seeds of *C. sativa* from 7.0 to 7,9 g of H_2_O/g of seed (*p*-value of Wilcoxon test: 0.011) while there is no difference between the control and the treated seeds of *A. thaliana* seeds. Figure 5 displays a summary of these different imbibition results in the case of the two kinds of seeds after 1, 6 and 24 h of imbibition.

#### 2.2.5. Lipidomic Analysis of the Seed Surface

**Figure 6:**
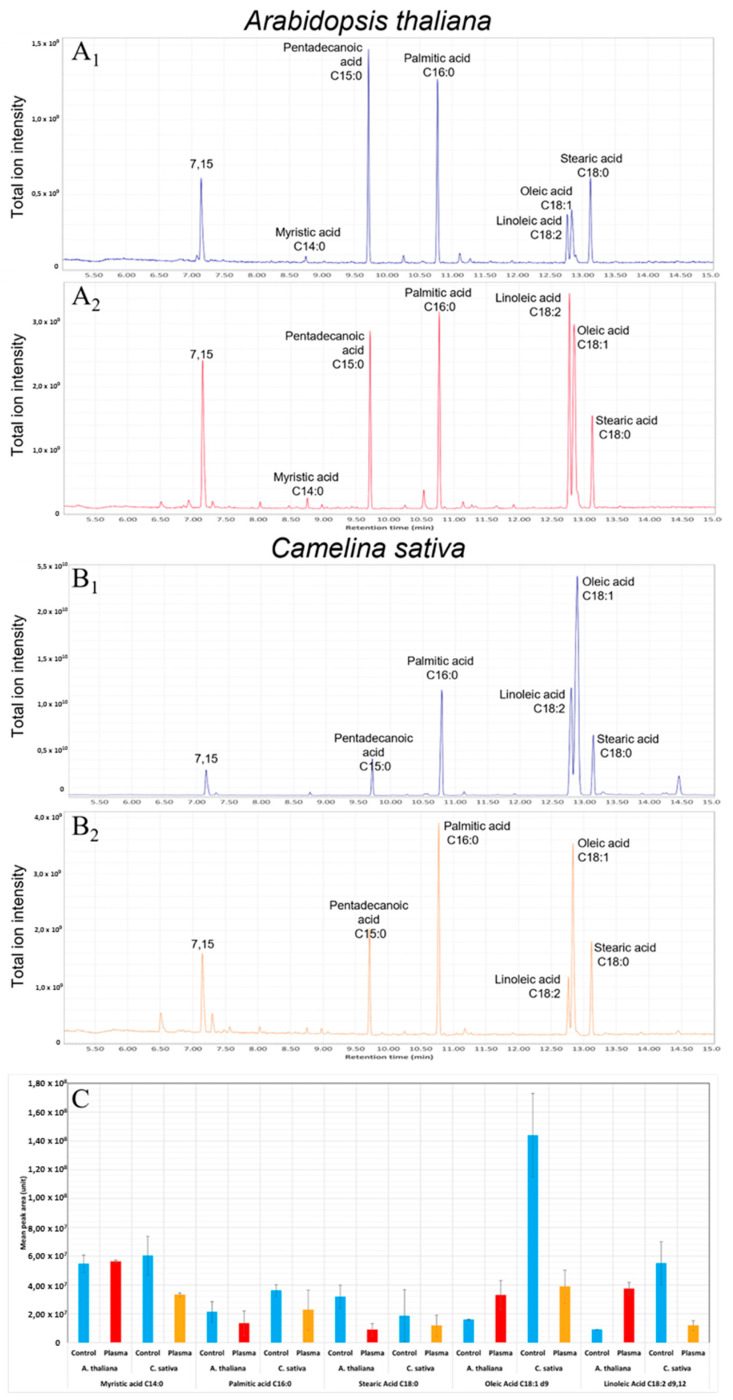
Change of free fatty acid composition on the surface of *A. thaliana* and *C. sativa* in the case of the control, the plasma seeds treated 1 min (*C. sativa*) and 15 min (*A. thaliana*). In blue, the analysis of the control seeds, in red, the treated *A. thaliana* seeds, and in orange, the treated *C. sativa* seeds. (**A**) GC/MS spectra of *A. thaliana* extract, (**A_1_**) control seeds, (**A_2_**) 15 min plasma treated seeds. (**B**) GC/MS spectra of *C. sativa* extract, (**B_1_**) control seeds, (**B_2_**) 1 min plasma treated seeds. (**C**) analysis of three samples, the relative abundance is calculated as a function of the C15 proportion used as a standard. All unsaturation places are noted as follows: “d”.

The seed surface compounds are analyzed in the case of both seeds (*A. thaliana* and *C. Sativa*) plasma treated and not (Figure 6). The free lipids of the surface of the seeds were solubilized with chloroform and analyzed using GC/MS diagnostics tools. The analyses are carried out in triplicate and standards are injected to identify the observed compounds. The treated seeds are therefore displayed in parallel with the control seeds for a comparison of the surface lipid composition.

Significant changes in the composition of free fatty acids extracted from the seed surface can be observed between treated and control seeds (Figure 6).

For *A. thaliana*, there is a reduction of palmitic (C16:0) and stearic (C18:0) saturated acids in the extracts obtained from treated seeds. While an increase of oleic (C18:1) and linoleic (C18:2) unsaturated acids is also noted, passing for C18: 2 from 9.0 × 10^6^ to 3.7 × 10^7^.

For the plasma treated *C. sativa*, the quantity of both saturated and unsaturated fatty acids decreases in all analyzed compounds passing for instance for oleic acid from 1.4 × 10^8^ to 3.8 × 10^7^.

Firstly, the higher concentration of oleic acid of *C. sativa* compared to *A. thaliana* agrees with the usual interest of this seed in agronomy.

Then, the observed changes of the lipids of both seeds can be correlated with the modifications of the seed surface morphology shown by SEM as well as the reduction of the seed imbibition underlined by contact angle and water uptake experiments.

## 3. Discussion

This study highlights some effects of DBD ambient air plasma treatments on two seeds of the same Brassicaceae family: *A. thaliana* and *C. sativa*. First, an increase in the germination rate and speed are observed for the plasma treated seeds of the two kinds. However, it is noteworthy that the optimal plasma treatment time depends on the considered seed. This optimal treatment time is evaluated thanks to both increase in the maximum germination (Gmax) rate and the germination speed (T50). The combination of a positive effect for both parameters (Gmax and T50) allowed the determination of the optimal treatment time. For *A. thaliana*, the optimal time with the ambient air DBD plasma device is 15 min while for *C. sativa* seeds it is 1 min. We therefore note a time-dependent effect on the plasma treatment which was already underlined in the literature [21,22,23]. The difference in the optimal treatment times may be due to the difference in biochemical composition between the two types of seeds. Indeed, the *C. sativa* seed is used in agronomy for its high fatty acid content [24]. The size of the seeds could also have an impact on the inter-electrode distance and therefore influence the physio-chemical reactions which occurred during the formation of the plasma [25]. Reactive oxygen and nitrogen species (RONS) are known to be essential during germination [26,27] but also to have a dose-dependent effect that can negatively affect the development of various plants [28]. As is known, low temperature plasmas generate many reactive species of oxygen and nitrogen (RONS) [29], so it is clear that such RONS species can have a positive effect on early germination steps [30] but only for the optimal exposure time to plasma, or in other words, for the optimal plasma dose. Indeed, for lower plasma doses, germination rate and speed are not efficient enough and for higher doses exceeding the optimal one, there are deleterious or inhibitory effects on the germination.

Furthermore, for *C. sativa* seeds, cotyledon area is increased for treated seeds. This can be due to the faster germination observed after treatment which allows faster and more advanced growth. It is probable that more particularly the produced RONS and maybe also the charged particles with plasma electric field applied during the optimal time certainly act as a stimulant allowing rapid growth during the early stages of germination (e.g., [31]).

SEM images highlight the appearance of a smooth structure on the surface of the seeds. Moreover, it appears that the level of the surface deformation is correlated with the treatment time as already shown elsewhere [32,33]. This seed surface modification could affect the germination speed while being synergistic with other stimulations. Indeed, after 1 min of treatment on *C. sativa* seeds, it appears that there is no significant change in the surface despite the increase of germination rate and speed.

Subsequently, experiments on the contact angle were carried out to observe the behavior of the surface of the treated seeds by the DBD ambient air plasma. The large reduction of the contact angle means an increase in the wettability of the seeds. This is in agreement with previous studies showing increases in the hydrophilicity of the seeds after different plasma treatments [34]. This is also in agreement with our water uptake experiments that showed a better imbibition between the control seeds and the plasma treated seeds. Indeed, after for instance, 6 h of imbibition, an increase of 24% of imbibition efficiency is observed in the case of *A. thaliana* and 11% in the case of *C. sativa*.

Afterwards, the permeability to tetrazolium salts was tested. It is noted that independently of the treatment times the permeability of the seeds is reduced. A previous study has shown that plasma treatment can minimize the effects of saline stress in the direct environment of the seeds during germination [19], and this could be explained by a decrease in salt permeability. This may appear in contradiction with the present experiments of water uptake and also with literature studies showing an increase in water uptake [13,35,36]. However, these imbibition studies are performed with water and not with saline solution which certainly explains this discrepancy.

In addition, biochemical modification of the surface of the two seeds was observed using GC/MS analysis. This resulted in a significant reduction of free fatty acids for *C. sativa* and some contrasted changes on the surface of *A. thaliana* showing an increase of unsaturated fatty acids and a decrease of saturated ones. This is not in disagreement with the SEM observations of the plasma smoothed surfaces interpreted as an etching and a macromolecular reorganization of the seed surface. Moreover, a recent study shows some changes due to the plasma on the wax at the surface of *Lavatera thuringiaca* seeds such as oxidation or nitration of surface compounds [20]. Similarly, a study on the soybean seeds has shown a certain abundance of C16 and C18 lipids on the seed surface and differences in imbibition as a function of different treatments to remove waxes [37]. Moreover, the present reorganization of the free lipids observed on the surface certainly influences the germination stimulation of the treated seeds via some processes of oxidation that could lead to a weakening of the macromolecular bond of the surface correlated with a better interaction with the water in the direct environment. This can be more true for saturated fatty acids that have a higher hydrophobicity and are lowered by the plasma treatment which directly improves the treated seeds’ imbibition and consequently their germination compared to the controls. Furthermore, in the case of treated *A. thaliana*, the rise of unsaturated acids that are very reactive probably contribute in one way or another to the neutralization of the plasma RONS effects thus requiring a longer plasma exposure time (15 min for optimal time) to reach the best germination rate and speed. While in the case of *C. sativa*, 1 min is enough to reach the best germination since more particularly the unsaturated linoleic acid becomes lower in the treated *C sativa* (1.2 × 10^7^) compared to the treated *A. thaliana* (3.7 × 10^7^).

The observed changes of the surface lipids during the plasma treatment result from a series of complex bio-chemical processes occurring on the seed surface, thus leading to a better germination rate and speed.

## 4. Experimental Set Up, Materials and Methods

### 4.1. Plasma Set Up

**Figure 7:**
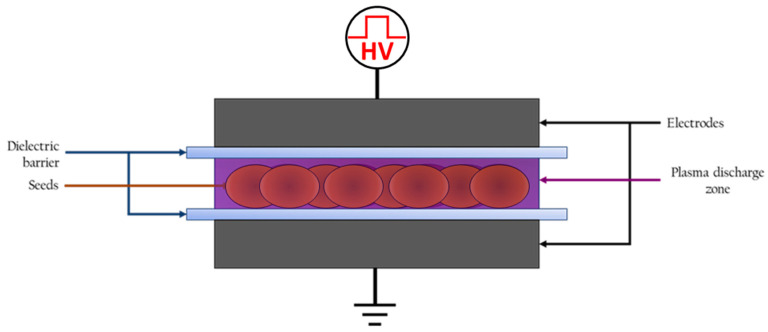
Scheme of the dielectric barrier discharge (DBD) plasma device. The purple depicts the plasma discharge zone generated between the two electrodes; the seeds are in direct contact with the plasma.

The present plasma device is a homemade dielectric barrier discharge (DBD, Figure 7). The produced low temperature plasma is generated directly in the ambient air. The electrodes (powered high voltage (HV) and grounded ones) are a stainless-steel cylinder covered by a glass of 1-mm thickness. The gap distance between HV electrode and the grounded one can be adjustable depending on the seed size. Seeds are placed directly over the lower glass in the gap between electrodes, where the plasma discharge is ignited, in contact with the glass tube and plate.

The pulsed power supply used to ignite the plasma is a positive mono-polar pulse voltage source. The electrical parameters were fixed based on our previous works [18,19] as follows: Voltage: 10 kV; Frequency: 10 kHz; Pulse duration: 1 µs.

### 4.2. Plasma Treatment

In this study, only the effects of direct treatment of DBD plasma on seeds were observed. For the plasma treatment, seeds were uniformly distributed between electrodes (Figure 7). Under these conditions, the plasma is ignited in the spaces between the two electrodes and covers all seeds surfaces. The seeds were directly exposed to the plasma with different treatment times between 1 to 15 min. Following the plasma treatment, various behaviors related to seed germination and development were observed.

### 4.3. Biological Material

**Figure 8:**
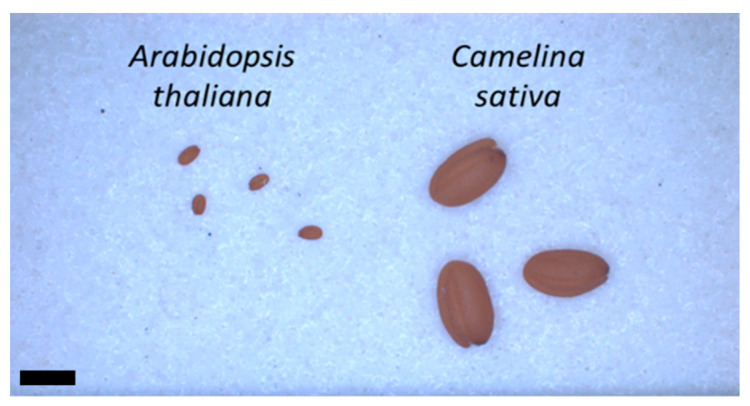
*A. thaliana* and *C. sativa* seeds. The scale bar is 1 mm.

The *Arabidopsis thaliana* and *Camelina sativa* seeds, both belonging to the Brassicaceae family, are used to emphasize the effects of the DBD air plasmas. The Brassicaceae family has a number of advantages for plasma treatment, including a large number of seeds per plant and a seed size small enough to allow plasma DBD treatment, (Figure 8). All those advantages allow a link to fundamental and agronomical research.

### 4.4. Germination Tests

There are several methods in the literature to measure the germination process by using various mathematical expressions to estimate, as emphasized by Ranal et al. [38], the germability or the germination time or the germination rate, etc. In the present work, we chose to observe first the testa and endsoperm ruptures and also the area of the cotyledons. Then, we estimated the parameters Gmax and T50, already used elsewhere [13], to know respectively the maximal percentage of germination (Gmax) at the end of observations and the time (T50) needed to reach 50% of maximum germination. The first parameter is representative of the germination rate and the second one of the germination speed.

The germination tests are carried out in Petri dishes of 35-mm diameter where is placed Whatman™ paper (3MM chromatography paper: mean thickness: 0.34 mm). The paper is upstream soaked with 0.5 mL of deionized water. The boxes are then sealed with ANAPORE adhesive to maintain maximum humidity while allowing gas exchange. Germination takes place in a culture chamber under fixed conditions at 40% humidity and continuous light (between 45 to 50 µE) with a photoperiod of 16 h (day)/8 h (night) at 22° C/20° C. Ruptures of the endosperm and testa are considered as the first outside signs of the beginning of germination. After plasma treatment, between 150 and 300 seeds of *A. thaliana* or 30 seeds of *C. sativa* are distributed per box. Untreated seeds are used as a control for comparison with plasma-treated seeds. Each experiment was repeated at least three times independently. Seed germination was followed for 80 h. The effects of plasma on late germination events were also observed thanks to area quantification of cotyledon. For this, only the seeds of *C sativa* are used because the size of *A. thaliana* sprout and the germination method does not allow a good differentiation between samples for those seeds. For *C. sativa*, the Petri dishes are scanned using a drawer scanner Microtek object scan 1600 with Scan wizard graph C1.09, then the images are analyzed using the ImageJ software to obtain a measurement of the cotyledon area.

### 4.5. Seed Surface Analysis

To analyze the effects of plasma treatment on the surface of the seeds, the following independents techniques were used.

#### 4.5.1. Scanning Electron Microscopy

The surface modifications of *A. thaliana* and *C. sativa* are observed using scanning electron microscopy images. The seeds are fixed to the surface of an aluminum bead using carbon tape and a spray of 10 nm of platinum is applied on the surface. The scanning electron microscope (SEM) used is a Quanta 250 FEG (FEI Company, Hillsboro, OR, USA). The parameters are set as follows: acceleration voltage: 5 kV, spot size: 3.0, pressure: 3.50 × 10^−4^ Pa. Observations are made on batches of about thirty seeds each, treated with air plasma DBD device during two processing times and also on equivalent batches of control seeds.

#### 4.5.2. Contact Angle

Subsequently, contact angle of a water droplet on the seed surface was measured. Due to size limitation, these tests were only performed on *C. sativa* seeds. Some 2- to 3-mm seed length allows the feasibility of this experiment. The seeds that are treated with DBD plasma 1 or 15 min, or the control seeds are placed one by one on a support provided with double-sided tape in order to fix them. A droplet of 0.3 µL of H_2_O is deposited using a 0.2 to 1 µL Eppendorf^®^ pipette. Photos and videos of the seeds are taken before, during and after the deposit of the drop. The images are acquired with a picamera V2 (8M pixels color camera) mounted on a Raspberry Pi 2B. Movies are captured at 90 fps in 640 × 480 pixels format for 7 s. The lens is adjusted to be approximately 1 cm from the seed resulting in a pixel size of 5.065 µm. Contact angle monitoring is performed using ImageJ^®^ software and Duncan’s test are performed to observe the significatively of the changes.

#### 4.5.3. Permeability Tests

The seed permeability test of *A. thaliana* and *C. sativa* was performed using triphenyltetrazolium chloride 2,3-5 (tetrazolium red, Sigma Aldrich, St. Louis, MO, USA). This salt has the property of being reduced to red formazan when it crosses the plasma membrane. 10 mg of control seeds and low temperature plasma treated seeds were immersed in 1.5 mL of 1% aqueous solution of tetrazolium red and incubated for 40 h at 28 °C. The seeds are then rinsed with distilled water three times in order to remove all the remaining reagent in the solution and crushed in 1.5 mL of 95% ethanol using 3 steel balls in a grinder Retsh ball for 3 min at 30 tr·s^−1^. After centrifugation at 3500 rpm during 3 min, the amount of formazan contained in the supernatant was evaluated by measuring the absorbance at 492 nm. It is noteworthy that usually the spectrophotometric determination of absorbance of the present formazan extracts was done at 485 nm (e.g., Vishwanath et al. [39]). However, a 492 nm filter was used in our experiments because it is the closest one that we have. This is an acceptable choice since this filter, having 10 nm of full width at half maximum, keeps a correct transmission for the studied absorbance. Anyway, the absorbance is correlated with the seed permeability. The test was repeated at least three times independently.

#### 4.5.4. Water Uptake

An experiment of water uptake is conducted on *C. sativa* and *A. thaliana.* Known quantities (between 0.0200 and 0.0400 g) of control and plasma-treated seeds were incubated in 1 mL of water. After 1, 6 or 24 h, the water is removed, and the seeds are weighed to highlight the absorption of water by the seeds. Those experiments were conducted using a balance Mettler AE200 ± 0.1 mg.

#### 4.5.5. Surface Lipids Analysis

To determine the nature of the surface modifications, lipid analysis was carried out for control and treated seeds. First, 100 mg of seeds, plasma treated or not, are placed in a glass tube with 1 mL of chloroform to solubilize the surface lipid compounds. The tubes are vortexed for 1 min, then the chloroform is recovered. After evaporation of the chloroform, 0.5 mL of BF_3_ methanol is added (Boron trifluoride-methanol, Sigma-Aldrich) then the tubes are incubated for 5 min at 60 °C. 1 mL of water is added to stop the reaction of BF_3_. The lipids are extracted with 1 mL of diethyl ether (step repeated three times), the tubes are then dried using a nitrogen fountain. Once completed, the tubes are dried and then taken up in 0.1 mL of 50% heptane-toluene mixture.

The samples (1 µL) are analyzed by GC/MS (Gas Chromatography/Mass Spectrometry), using a TSQ quantum thermo scientific analyzer with a “full scan” type in spitless mode. The identification of the fatty acids is confirmed with FAME Standard. GC separation was performed on a DB5MSi column (30 m × 0.25 mm, 0.25 μm film thickness; Phenomenex, Hongkong, China). The temperature of split/splitless injector was 230 °C and helium was carrier gas at constant flow (1 mL/min). Temperature program used was: 100 °C for 2 min, then 20 °C/min until 190 °C, 6 °C/min until 270 °C, followed by 15 °C/min until 340 °C, and finally held at 340 °C for 2 min. The mass spectrometry analyses were performed on positive mode. Molecules were ionized by electron impact ionization (EI, 70 eV, ion source temperature: 250 °C) and data acquisition was performed on full scan.

The qualitative analysis was performed using Xcalibur software and quantitative analysis using Trace Finder software (Thermo Scientific, Waltham, MA, USA).

### 4.6. Statistical Analyses

Data analysis was performed using R studio V1.0.136 (cran.r-project.org, access on 7 September 2021) and individual data were plotted and expressed as mean ± standard error to the mean. The significant differences were determined using the Duncan test or Wilcoxon test.

## 5. Conclusions

This research work is aimed to determine some effects of low temperature plasma treatment on the early stages of germination of two Brassicaceae seeds and to shed light on the origin of those effects. The considered low temperature plasma is an ambient air plasma generated by a dielectric barrier discharge device using a pulsed power supply. Plasma seed treatments based on various exposure times and several analyses are carried out on the seeds of *A. thaliana* and *C. sativa* to better understand the improvement of germination speed and rate. It is shown as expected that the obtained optimal exposure time is seed-dependent, the optimal time being the plasma exposure time allowing the best increase of both the rate and the speed of germination. Thereby, this optimal time is 15 min for *A. thaliana* with an increase of about 85% compared to the control after 48 h of germination and 1 min for *C. sativa* with an increase of about 75% compared to the control after 32 h of germination. It is evidenced that such plasma-stimulated germination must be linked to the major biochemical structural changes that occurred at the surface of the seed after the plasma treatment. This visual change is demonstrated by scanning electron microscopy analysis but also by analyzing the permeability of seeds to salts or using the contact angle or the water uptake to estimate the wettability. About this later parameter, it is shown after 6 h of seeds imbibition, an increase of 24% of imbibition efficiency due to the plasma treatment in the case of *A. thaliana* and around 11% in the case of *C. sativa*.

Furthermore, the lipidomic analysis has shown for *C. sativa* a decrease of saturated (palmitic and stearic) and unsaturated (oleic and linoleic) fatty acids while for treated *A. thaliana* there is a decrease of saturated fatty acids and an increase of the unsaturated ones. It is noteworthy that for seeds of both species, there is a marked decrease of saturated fatty acids, known to be hydrophobic. Therefore, such changes of the surface lipidic compounds are coherent with the surface changes shown by the previous SEM pictures or wettability or permeability analysis. It is therefore suggested that these surface changes are strongly involved in the improvement of the plasma stimulated germination. In addition, the rise in the case of treated *A. thaliana* of unsaturated acids that are very reactive is probably at the origin of the neutralization of the plasma RONS effects thus leading to a longer plasma exposure time (15 min for optimal time) to reach the best germination. While in the case of *C. sativa*, 1 min is enough to reach the best germination since the unsaturated linoleic acid becomes lower in the treated *C sativa* (1.2 × 10^7^) compared to the treated *A. thaliana* (3.7 × 10^7^).

Finally, all the observed bio-chemical changes on the seed surfaces are obviously related to their exposure more particularly to the numerous plasma reactive species of oxygen and nitrogen and probably also to charged particles and plasma electric field.

## Data Availability

Not applicable.
